# A new method for quantitative detection of Lactobacillus casei based on casx gene and its application

**DOI:** 10.1186/s12896-019-0587-6

**Published:** 2019-12-10

**Authors:** Xiaoyang Pang, Ziyang Jia, Jing Lu, Shuwen Zhang, Cai Zhang, Min Zhang, Jiaping Lv

**Affiliations:** 1 Beijing Advanced Innovation Center for Food Nutrition and Human Health, Beijing Technology & Business University (BTBU), Beijing, 100048 China; 2 Institute of Food Science and Technology, Chinese Academy of Agricultural Science, Beijing, 100193 China; 3 Laboratory of Environment and Livestock Products, Henan University of Science and Technology, Luoyang, 471023 China

**Keywords:** TaqMan MGB RT-PCR, Lactobacillus casei, Cas, Rapid detection method, 16 s rRNA

## Abstract

**Background:**

The traditional method of bacterial identification based on 16S rRNA is a widely used and very effective detection method, but this method still has some deficiencies, especially in the identification of closely related strains. A high homology with little differences is mostly observed in the 16S sequence of closely related bacteria, which results in difficulty to distinguish them by 16S rRNA-based detection method. In order to develop a rapid and accurate method of bacterial identification, we studied the possibility of identifying bacteria with other characteristic fragments without the use of 16S rRNA as detection targets.

**Results:**

We analyzed the potential of using *cas* (CRISPR-associated proteins) gene as a target for bacteria detection. We found that certain fragment located in the *casx* gene was species-specific and could be used as a specific target gene. Based on these fragments, we established a TaqMan MGB Real-time PCR method for detecting bacteria. We found that the method used in this study had the advantages of high sensitivity and good specificity.

**Conclusions:**

The *casx* gene-based method of bacterial identification could be used as a supplement to the conventional 16 s rRNA-based detection method. This method has an advantage over the 16 s rRNA-based detection method in distinguishing the genetic relationship between closely-related bacteria, such as subgroup bacteria, and can be used as a supplement to the 16 s rRNA-based detection method.

## Background

In recent years, many studies have confirmed that intestinal flora is associated with a variety of nutritional and metabolic diseases such as obesity [1, 2] and type 2 diabetes [3, 4]. In the field of scientific research, the study of intestinal flora has become of utmost importance. Billions of bacteria in the intestine live in symbiosis with each other for the host’s nutritional and metabolic needs [5, 6]. The intestinal flora of the host have a close relationship with the storage and absorption of nutrition [7, 8], immunity [9], as well as the regulation of sRNA regulation [10]. Through their genes, intermediates and metabolites, these florae affect the host’s nutritional absorption, metabolism, weight, immunity, and several other aspects [11, 12]. Once the balance of intestinal flora is disrupted, a variety of nutritional and metabolic symptoms appears in the host [13, 14]. Although intestinal florae have been shown to be associated with many metabolic diseases, a lot of work still needs to be done in order to establish the differences between related and casual diseases.

Current research on intestinal flora are mostly based on Illumina’s high-throughput sequencing technology; which has the advantages of high throughput, short time, and low cost [15, 16]. However, its low resolution characteristic is a big drawback, and most bacteria can be identified only at genus level. Consequently, at the species level only a few bacteria can be identified using the technology, with an inability to distinguish intestinal flora among sub-species or strains. In fact, the roles of different species of the same genus in a host are remarkably different. For instance, studies have shown that different species of the same genus or family exhibit variations to increase or decrease during weight gain in high-fat-fed animals [17, 18]. Obviously, their relationships with the development of obesity cannot be fully elucidated. It is presumed that, while some of the intestinal bacteria are related to obesity, others are not. This suggests that it is necessary to establish a more suitable method with higher resolution to study the relationship between intestinal florae and their hosts. The search for specific gene fragments from the target bacterial genome, and the development of a corresponding detection method, could be the key factor to solve this fundamental problem.

The common strategy for the search of specific fragments of bacteria involves the analysis of bacterial 16 s rRNA sequencing, and then find the specific fragments from its variable area [19, 20]; However using this method, it is sometimes difficult to distinguish closely related bacteria such as *L. casei* and *L. rhamnosus*, because of the 99% similarity in the 16 s rRNA whole sequences (1540/1558). Due to the fact that a specific bacteria fragment from the 16 s rRNA sequence is difficult to find, it is necessary to search for new characteristic fragments from other areas of the bacterial genome. In this study, we found that some CRISPR-associated proteins (Cas) are strain-specific and could be used as target gene fragments for the identification of strains. The bacterial identification based on *casx* gene, could be used as an supplement to the conventional method based on 16 s rRNA.

CRISPR is a special-function DNA sequence that widely exists in bacteria and archaea genomes [21, 22]. The sequence covers one leader, multiple short and highly conserved repeats, as well as multiple spacers. CRISPR is considered to be the bacteria’s immune system [23, 24]. After the bacteria are infected by a virus, the surviving bacteria can capture a characteristic DNA fragment from the virus and then integrate it into their genome CRISPR area. At a subsequent viral invasion, the bacteria can quickly identify them according to the CRISPR archive area and then activate the endonuclease to cut the invading virus; equivalently acting as immunity to the virus. Each time a new virus is encountered, the bacteria can capture its characteristic DNA fragments and insert them into their own CRISPR area. The above functions of bacteria are performed by a series of CAS proteins. Although some *cas* genes (such as the widely known *cas*9 gene), have great similarities in sequences among different bacteria, several others have low similarities. We selected all the *cas* genes annotated on the genome of *Lactobacillus casei* and then aligned them with their corresponding genes of ten *Lactobacillus* strains. The results showed that a *casx* gene in the flanking sequence of CRISPR had lower similarity with other *Lactobacillus* species. Primers and probes for fluorescence quantitative polymerase chain reaction (qPCR) were designed according to the *casx* gene. Furthermore, the results also showed that *L. casei* from other intestinal microbes could be accurately distinguished with high sensitivity and reproducibility using this method. In this study, the bacteria from a large microbial flora were accurately identified and their abundance detected using fluorescence qPCR assay based on the *casx* gene of *L. casei.* The method is high sensitivity and repeatable. This study established the foundation for the study of the relationships between intestinal microbes and their host via species or subspecies.

## Results

### The acquisition of *Lactobacillus casei* specific gene fragments

**Fig. 1:**
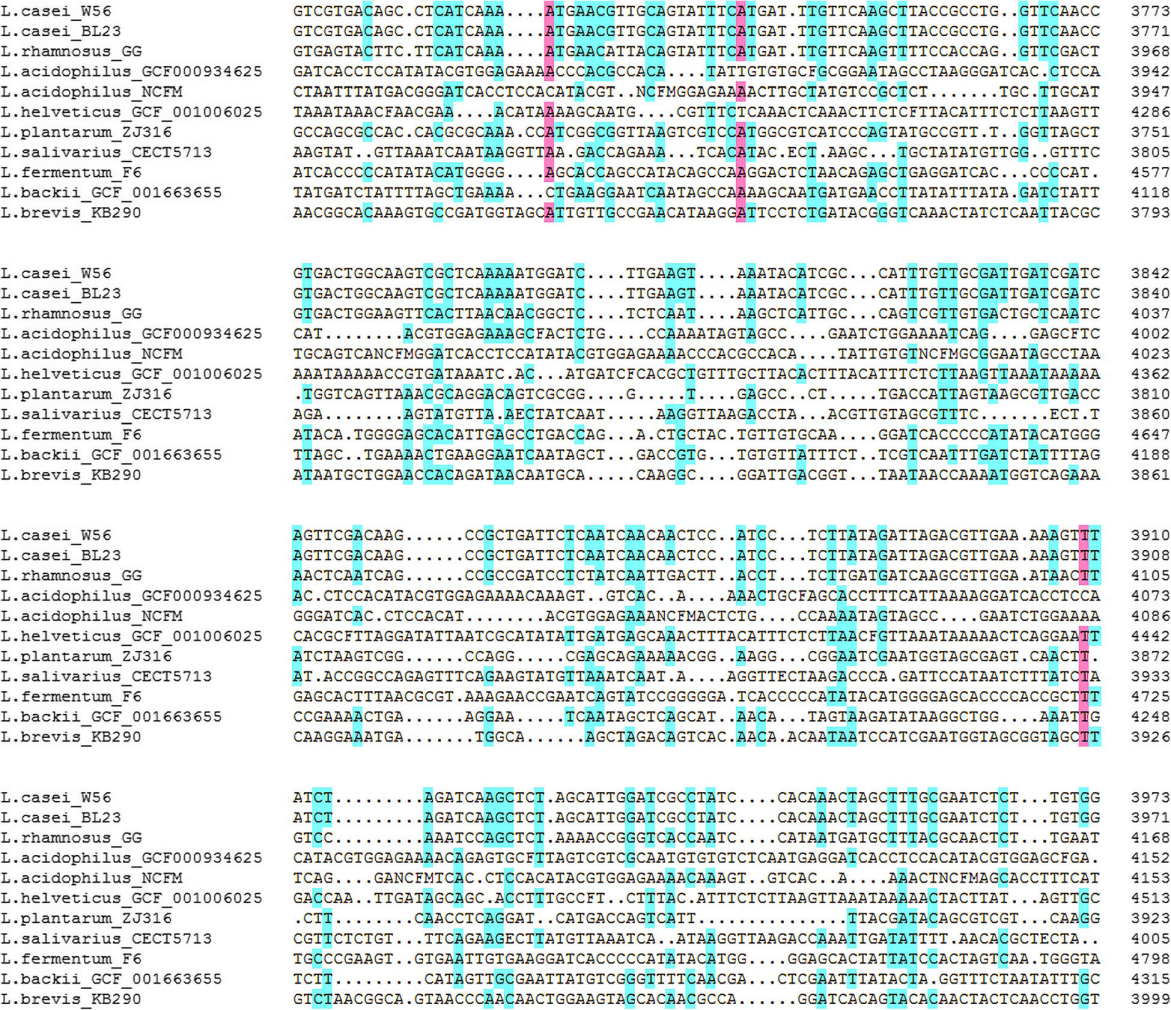
The alignment result of the Lactobacillus CRISPR flanking sequence

The CRISPR sequences obtained from this study are shown in Table 1. We compared the CRISPR flanking sequence of *L. casei* with other strains of *Lactobacillus*, and found that one *casx* gene had a conserved region of ~ 270 bp (Fig. 1). The two *L. casei* strains in this region had an identical gene sequence (*L. casei* w56: 2325395–2,325,664; *L. casei* BL23: 2328749–2,329,018), and was quite different from other *Lactobacillus* species. Although *L. rhamnosus* is closely related to *L. casei*, the *casx* gene of *L. rhamnosus* is different from that of *L. casei*. Therefore, this region could be used as a candidate target gene for the detection of *L. casei.* In order to verify the specificity of this gene, the 270-bp *casx* gene fragment was obtained by Blast in the Genbank database. The results showed that the fragment had high similarity with the sequence of the six strains in the genome and Genbank database, and all six strains were *L. casei*; indicating the species specificity of the sequence.

**Table 1:** CRISPR distribution of Lactobacillus

Strain	Genbank No.	DR consensus	Position
Lactobacillus acidophilus GCF_000934625	NZ_CP010432.1	GGATCACCTCCACATACGTGGAGAAAAT	1,541,318–1,543,298
Lactobacillus acidophilus NCFM	NC_006814	GGATCACCTCCACATACGTGGAGAAAAT	1,541,039–1,543,019
Lactobacillus backii GCF_001663655	NZ_CP014623	GATCTATTTTAGCTGAAAACTGAAGGAATCAATAGC	844,462–846,673
Lactobacillus brevis KB290	NC_020819	GTATTCCCCACACATGTGGGGGTGATCC	1,071,990–1,072,505
Lactobacillus casei W56	NC_018641	GCTCTTGAACTGATTGATTCGACATCTACCTGAGAC	2,323,692–2,325,115
Lactobacillus casei BL23	NC_010999	GCTCTTGAACTGATTGATTCGACATCTACCTGAGAC	2,327,048–2,328,469
Lactobacillus delbrueckii subsp. bulgaricus ATCC 11842	NC_008054	GTATTCCCCACGCAAGTGGGGGTGATCC	764,071–766,562
Lactobacillus fermentum F-6	NC_021235	GGATCACCCCCATATACATGGGGAGCAC	1,348,092–1,352,667
Lactobacillus helveticus GCF_001006025	NZ_CP011386	CTTTACATTTCTCTTAAGTTAAATAAAAAC	1,644,308–1,647,001
Lactobacillus plantarum ZJ316	NC_020229	GTCTTGAATAGTAGTCATATCAAACAGGTTTAGAAC	359,930–360,361
Lactobacillus rhamnosus GG	NC_013198	GCTCTTGAACTGATTGATCTGACATCTACCTGAGAC	2,265,855–2,267,474
Lactobacillus salivarius CECT 5713	NC_017481	GTTTCAGAAGTATGTTAAATCAATAAGGTTAAGACC	121,320–123,253

### Fragment-specific validation results

According to the specific fragment in this study, the primers for fluorescence qPCR were designed and named as 06232F and 06232R, while the probe match for the primers was designed and named as 06232P. The probe was linked to a luminophore FAM on the 5′ end and a quencher MGB-NFQ on the 3′ end. The details of the primers and probes are presented in Table 2.

**Table 2:** Amplification primers and Taqman–MGB probes used for specific detection

Primers or probes	Nucleotide sequence(5′ → 3′)	Amplicon size
Primers		
06232F	TCAACCGTGACTGGCAAGT	91
06232R	AGCGGCTTGTCGAACTGA	
M13–47	CGCCAGGGTTTTCCCAGTCACGAC	247
M13–48	AGCGGATAACAATTTCACACAGGA	
Probes		
06232P	FAM-CTCAAAAATGGATCTTG-MGB-NFQ	

**Fig. 2:**
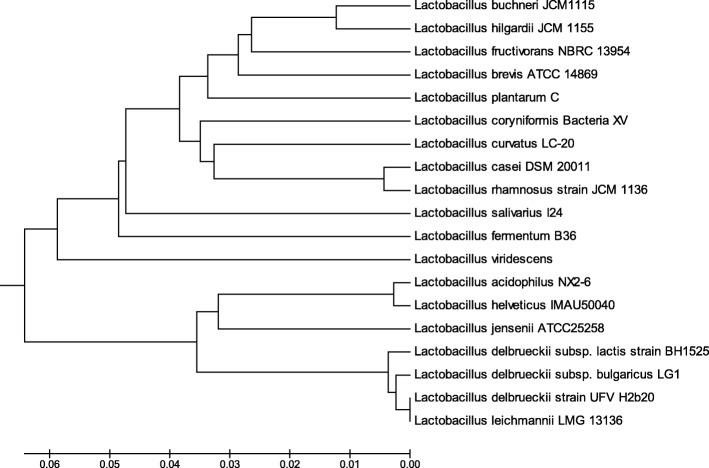
Phylogetic analysis on 16S rRNA of 19 Lactobacillus strains

**Fig. 3:**
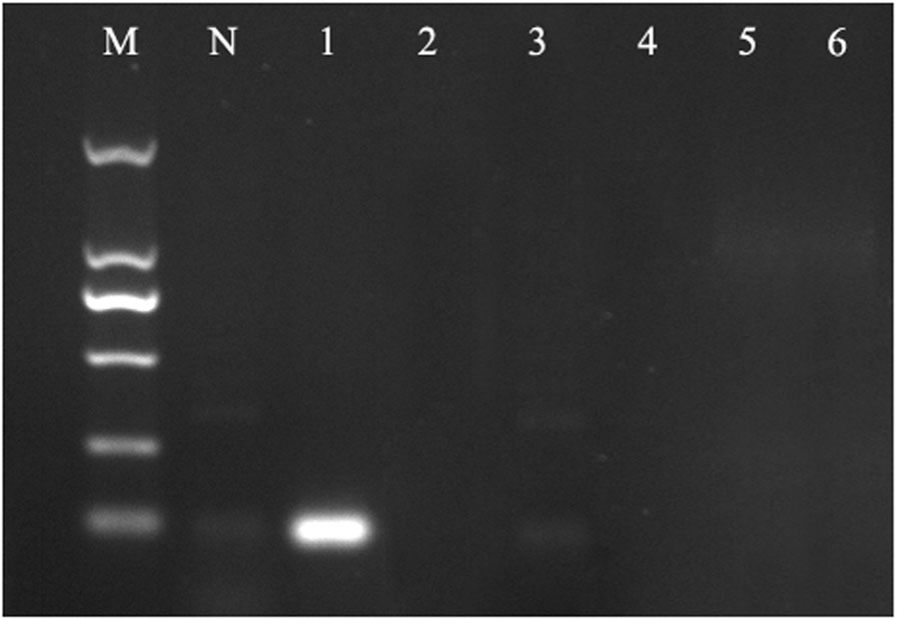
Primer specificity validation results. M: D2000 marker (TIANGEN, MD114); N: Negative control; 1: Lactobacillus casei SY13; 2: L. plantarum M15; 3: L. curvatus znj160802; 4: L. coryniformis znj160401; 5: L. rhamnosus YL4; 6: L. brevis znj160202

The genetic relationship of 19 *Lactobacillus* strains was analyzed. The results show that *L. casei* was closely related to *L. brevis*, *L. plantarum*, *L. curvatus*, *L. coryniformis*, and *L. rhamnosus* (Fig. 2). Therefore, six strains of *Lactobacillus* (*L. casei* SY13, *L. plantarum* M15, *L. curvatus* znj160802, *L. coryniformis* znj160401, *L. rhamnosus* YL4, and *L. brevis* znj160202) were selected and their genomes extracted. The genomes of the six strains were amplified by PCR with 06232F and 06232R primers. As a result, the target fragment of about 90 bp was obtained from *L.casei* SY13 genome and no target fragment was obtained from the genomes of other bacteria (Fig. 3). This indicated that the specificity of the primers was good.

### Establishment of the fluorescent qPCR detection method

A 93 bp DNA fragment was amplified from the genome of *L. casei* SY13 using the 06232F and 06232R primers. The fragment was ligated with pMD19T plasmid and transformed into *E. coli* DH5α. Positive clones were screened on lysogeny broth plates which contained 50.0 μg/mL ampicillin, extracted and correctly sequenced. The target DNA of standard substance pMD19T-CS was assayed to determine its concentration using Spark 20 M Multiscan Spectrum. The concentration of pMD19T-CS was 30.05 ng/μL and the unit was converted to copies/μL according to formula:

$$ \mathrm{plasmid}\ \mathrm{concentration}\left(\mathrm{copies}/\mu \mathrm{L}\right)=\frac{\mathrm{plasmid}\ \mathrm{concentration}\left(\mathrm{ng}/\mu \mathrm{L}\right)\times 6.02\times {10}^{23}}{\mathrm{pMD}19\mathrm{T}-\mathrm{CS}\ \mathrm{length}\left(\mathrm{bp}\right)\times 660\mathrm{g}/\mathrm{mol}} $$

**Fig. 4:**
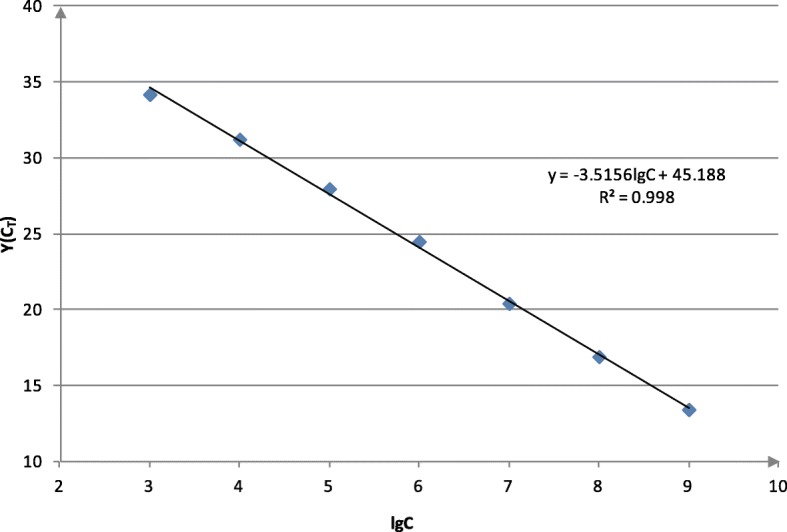
Standard curve between concentrations of standard DNA and C_T_ value

The DNA standard was diluted from 10^3^ to 10^8^ copies/μL and used to generate the standard curve (Fig. 4). The regression equation was:

$$ \mathrm{Y}=-3.53\mathrm{lgC}+45.28 $$

Where R^2^ = 0.998, Y represents C_T_, while C represents the concentration of standard DNA. The efficiency of amplification was 92.011% and the detection limit was 10^2^ copies/μL.

### Analysis of mice experiment

Balb/c mice were fed with *L. casei* SY13 for 7d, and then sacrificed at the end of the feeding trial. The content of the different parts of their intestines were analyzed to quantify *L. casei* SY13. The results showed that the target bacteria were not found in the intestinal tract of the negative control group, which implied that there were no endogenous *L. casei* in the intestines of the mice. However, the target bacteria were detected in the experimental group, and the highest numbers were found in the cecum. Interestingly, the target bacteria were not detected in the ileum (Table 3). The quantities of *L. casei* subsp. *casei* SY13 in the jejunum, cecum, and colon were 1.6 × 10^5^ copies/g, 2.1 × 10^6^ copies/g, and 1.7 × 10^6^ copies/g respectively. The results indicated that the fluorescence qPCR method based on the *casx* gene could specifically detect *L. casei* from the intestinal microbial flora of mice.

**Table 3:** Quantity of target bacteria in intestinal tract

	jejunum	ileum	cecum	colon
	C_T_			
Negative group	–	–	–	–
Experimental group	27.14 ± 0.79^a^	–	23.21 ± 1.06^b^	24.08 ± 1.08^b^

^ab^ Means in the same row different superscript letter differ significantly(*P* < 0.05)

## Discussion

The effort to search for the specific gene fragments of bacteria had plagued the researchers of environmental microbiology for a long time. In the past, the conventional strategy was to search the 16 s rRNA sequence and then select the conserved region sequence as the target fragment for detection [25–27]. However, this method is insufficient to distinguish closely related bacteria. The current microbiome technique involves the use of high-throughput sequencing technology based on the V3–V4 region of the 16 s rRNA of the bacteria, to distinguish the different bacteria in the sample [28]. Although this method identified most of the microorganisms in the sample, however it cannot distinguish bacteria that are closely related in the same genus [29].

In order to identify bacteria more quickly and accurately, many researchers had explored several other options. For instance, multiplex PCR was used to detect *L. casei* [30] in such a way that two sets of primers were designed to ensure the specificity of *L. casei* ATCC 393 in the multiplex PCR system. However, this method was not able to quantify *L. casei* ATCC 393; thus it is usually combined with conventional microbiological cultivation. It is usually a difficult and labor-intensive procedure. FISH probe and hybridization were also used to detect *Lactobacillus*. Slides were made from intestinal-tract samples and examined using an Olympus BH2 epifluorescence microscope [31]. Without precision, the researchers visually recognized only the number of *Lactobacillus* that adhered to the intestinal tract. Thus, the development of a simple, highly efficient, and highly specific method was urgently required. We used the *casx* gene of *L. casei* and developed a method for the rapid and accurate detection of *L. casei* by fluorescence qPCR. The core of this method is to find the appropriate casx gene fragment on the flank of CRISPR. In order to verify the general applicability of this method, we tested it on *Legionella pneumophila*. *L. pneumophila* includes two main subspecies, one is *L. pneumophila* subsp. *fraseri*, the other is *L. pneumophila* subsp. *pneumophila*. Traditional methods based on 16S rRNA are difficult to distinguish these two kinds of bacteria. First of all, we searched for CRISPR region in the whole genome of *L. pneumophila* subsp. *fraseri* GCF_001886795_and *L.pneumophila* subsp. *pneumophila* GCF_001592705_respectively. The results are shown in Additional file 1: Table S1. Then, 3000 bp was taken from CRISPR’s flank as candidate sequence, the extracted sequences are shown in Additional file 2: Table S2. Using ClustalX 2.0 to align the extracted flank sequence. Based on the sequence alignment results, a 256 bp specific sequence was found on *L.pneumophila* subsp. *pneumophila* GCF_001592705, which only existed in *L.pneumophila* subsp. *pneumophila* GCF_001592705 genome, but not in *L. pneumophila* subsp. *fraseri* GCF_001886795, the sequence information is shown in Additional file 3: Table S3. In order to verify the specificity of the sequence, we used blast tool to align the 256 bp sequence in GenBank, the results are shown in Additional file 4: Figure S1. It can be seen from the results that the 256 bp fragment has good specificity and can distinguish *L.pneumophila* subsp. *pneumophila* from *L. pneumophila* subsp. *fraseri*. The above case in *L.pneumophila* can prove that the method provided in this study is not only applicable to *Lactobacillus casei*, but also applicable to other bacteria.

## Conclusions

In this study, we used the *casx* gene of *L. casei* and developed a method for the rapid and accurate detection of *L. casei* by fluorescence qPCR. *L. casei* and *L. rhamnosus* were easily distinguished with the use of this method. There is an extremely high similarity between the two bacteria in 16 s rRNA sequences, therefore, it is difficult to distinguish them from each other based on the 16 s rRNA method. The *casx* gene-based method of identification developed in this study was able to rapidly and accurately distinguish the two bacteria. Finally, we validated the accuracy and sensitivity of the method using mouse experiments. This method has an advantage over the 16 s rRNA-based detection method in distinguishing the genetic relationship between closely-related bacteria, such as subgroup bacteria, and can be used as a supplement to the 16 s rRNA-based detection method.

## Methods

### Bacteria strains, plasmids, and mice

The bacteria and plasmids used in this study are shown in Table 4. *Lactobacillus* strains were statically cultured in MRS broth (Cat. No. CM187, Beijing Land Bridge Technology Co., Ltd., China) at 37 °C. *Escherichia coli* DH5α was grown at 37 °C in LB broth (1.0% peptone, 0.5% yeast extract powder, 1.0% NaCl; pH 7.4), SPF BALB/c mice were purchased from Beijing Vital River Laboratory Animal Technology Co., Ltd. (Beijing, China).

**Table 4:** Bacterial strains and plasmids used in this study

Strain or plasmid	Relevant genotype or description	Reference and/or source
Strains Lactobacillus casei SY13	Isolated from milk cheese	Preserved in our lab
Lactobacillus plantarum M15	Isolated from natural fermented yak milk	Preserved in our lab
Lactobacillus rhamnosus YL4	Isolated from milk cheese	Preserved in our lab
Lactobacillus curvatus znj160802	Isolated from traditional Chinese fermented dairy products	Dairy Processing Laboratory, Beijing Technology and Business University
Lactobacillus coryniformis znj160401	Isolated from a goat’s milk cheese	Dairy Processing Laboratory, Beijing Technology and Business University
Lactobacillus brevis znj160202	Isolated from traditional Chinese fermented dairy products	Dairy Processing Laboratory, Beijing Technology and Business University
Escherichia coli DH5α	F^−^,φ80d lacZ △M15,△(lacZYA-argF)U169, deoR, recA1, endA1, hsdR17 (rk^−^,mk^+^), phoA, supE44, λ^−^, thi-1, gyrA96, relA1	TaKaRa
Plasmids		
pMD19T	clone vector; Amp^r^	TaKaRa
pMD19T-CS	pMD19T derived plasmid, DNA standard for fluorescence quantitative PCR	this study

### Acquisition and alignment of cas sequences of *Lactobacillus*

The CRISPR sequence of *Lactobacillus* was derived from the CRISPR database (http://crispr.i2bc.paris-saclay.fr). Due to the fact that some *cas* genes are not annotated in the genome of *Lactobacillus,* the CRISPR flank 3000 bp was selected as the analysis sequence to prevent the loss of some key *cas* genes. ClustalX 2.0 was used for the alignment of sequence.

### Design of primers and TaqMan-MGB probes

According to the alignment results, we searched for the characteristic fragment of *L. casei*. The primers and TaqMan-MGB probe to detect *L. casei* were designed by Primer Express 3.0 based on the characteristic fragment. The syntheses of primers and probes were entrusted to the Beijing Genomics Institute (BGI). In this study, the specificity of the characteristic fragment was verified from two procedures. Firstly, the characteristic fragment was subjected to Blast in Genbank to examine whether the sequence matched the bacteria other than *L. casei*. Secondly, 19 *Lactobacillus* strains were used to reconstruct a phylogenetic tree based on their 16S rRNA nucleotide sequences using MEGA 6.0. The five closely related strains to *L. casei* were selected to verify the specificity of the primers.

### Establishment of the fluorescent qPCR detection method

Bacterial genome DNA was extracted and purified using a nucleic acid extraction kit (Cat. No. DP302, TIANGEN Biotech, Beijing, China) according to the manufacturer’s instructions. Genome DNA was extracted from the intestinal contents and purified with the TIANamp Stool DNA Kit (Cat. No. DP328, TIANGEN Biotech, Beijing, China) according to the manufacturer’s instructions.

PCR was performed to obtain target DNA fragments using primers 06232F and 06232R. Target DNA fragments were recovered from agarose gels using the TIAN Gel Purification Kit (Cat. No. DP209, TIANGEN Biotech, Beijing, China). The target fragment was then inserted into the pMD19T vector (Cat. No. 6013, Takara Biomedical Technology (Beijing) Co., Ltd. Beijing, China). The ligation products were transformed into DH5α competent cells using the heat shock method. Positive clones were chosen and inoculated into the LB broth containing 100 μg/mL ampicillin and then cultured overnight in an incubator at 37 °C and 200 rpm. The TIANprep Plasmid Kit (Cat. No. DP103, TIANGEN Biotech, Beijing, China) was used to extract plasmids from the cultured bacteria suspension. The extracted plasmids were sequenced to verify the inserted fragments. Sequencing was entrusted to BGI. Sequencing validates the correct transformants as the DNA standard. The concentration of the standard DNA was detected on Spark 20 M Multiscan Spectrum (Tecan Group Ltd., Switzerland).

The DNA standard was used to generate a standard curve and analyze assay sensitivity. The DNA diluted from 10^2^ to 10^8^ copies/μL was used as a template to perform RT-PCR. A TaqMan-MGB probe was used to detect the C_T_ value. A standard curve between the C_T_ value, dilution gradient and linear regression equation was generated automatically by ABI 7500 Real-Time PCR System. The coefficient of determination (R^2^) value was also demonstrated. The DNA standard diluted from 10^0^ to 10^4^ copies/μL was tested to observe the detection limit.

### Mice experiment

After the Balb/c mice were treated with *L. casei* for 7.0 d, we collected the cecum and colon contents and extracted the genomic DNA, to further test the specificity of the primers and probes in the intestinal flora. The number of *L. casei* in different parts of the intestinal tract was measured by fluorescence qPCR using the extracted genomic DNA as templates. The mice fed without *L. casei* were used as negative control. Six male mice were used in this experiment. They were randomly divided into two groups; three in each cage. They were left for 7.0 d to adapt to their environment, and water and basal diets were freely given. At the onset of the treatment, the experimental group was gavage-induced with 10^9^ cfu *L. casei SY13*, while the negative group was administered sterile water. Mice were sacrificed 7 days after treatment. Carbon dioxide method was used to euthanize mice. The methods were referenced to previously published literature [32]. The sacrificed mice were dissected; the jejunum, ileum, cecum, and colon were extracted and preserved in liquid nitrogen. The genome DNA of the intestinal contents was also extracted. The quantity of the target bacteria was measured by RT-PCR. Data analysis was conducted using SPSS 20.0 (IBM Corporation, Armonk, NY, USA).

## Supplementary information

**Additional file 1: Table S1.** CRISPR region of *Legionella pneumophila*.

**Additional file 2: Table S2.** Flanking sequence of CRISPR region.

**Additional file 3: Table S3.** Specific sequence selected from sequence alignment results.

**Additional file 4: Figure S1.** The result of sequence BLAST.

## Data Availability

The datasets used and analyzed for the current study are available from the corresponding author upon reasonable request.

## Abbreviations

Cas proteins: CRISPR-associated proteins
QPCR: Quantitative Polymerase Chain Reaction
RRNA: Ribosomal RNA
RT-PCR: Realtime Polymerase Chain Reaction
