# Supplementary material for: A new method for quantitative detection of Lactobacillus casei based on casx gene and its application
Source: BMC Biotechnol. 2019 Dec 10;19:87. doi: 10.1186/s12896-019-0587-6 (PMC6902566; doi:10.1186/s12896-019-0587-6)
Supplement: Supplementary file 1 — Additional file 1: Table S1. CRISPR region of Legionella pneumophila. [file 12896_2019_587_MOESM1_ESM.docx]

Table S1 CRISPR region of *Legionella pneumophila*

| Legionella pneumophila subsp. fraseri GCF_001886795 | | | |
| --- | --- | --- | --- |
| RefSeq:NZ_CP017457 (chromosome circular) | | Number of repetitions :11 |  |
| Position | Repeat | Spacer | Position |
| 200766 | ATTTCATTAGTTAAATTTTTAGATGGGGGATTTTTGG | AAGAAGTCAGGCTACGACCATGAAAACGATGCGAC | 200837 |
| 200838 | GTTTCAGTGGTTGGATTTTTAGATGAGGGATTATTGG | TTTCTTTAAGCTTTTAAAACGCTTTTCACATACA | 200908 |
| 200909 | GTTTCAGTGGTTGGATTTTTAGATGAGGGATTATTGG | CTTTTAGCATTCGAGATTTTGATGATGAAATGGAAGT | 200982 |
| 200983 | GTTTCAGTGGTTGGATTTTTAGATGAGGGATTATTGG | TTCAAAGAAAGTTAATCCCGTTGAGTGGGTTAGGAT | 201055 |
| 201056 | GTTTCAGTGGTTGGATTTTTAGATGAGGGATTATTGG | ATACTTTGTGGGTTTTAGTAGAGGAGCCTTTCTTGTC | 201129 |
| 201130 | GTTTCAGTGGTTGGATTTTTAGATGAGGGATTATTGG | GTTGAGATCTTTTGAAGTAGTGGAAAAGGGGGATGT | 201202 |
| 201203 | GTTTCAGTGGTTGGATTTTTAGATGAGGGATTATTGG | TTAAGGATAAACAGACAAGCCTTTTTTGCTGAGAAA | 201275 |
| 201276 | GTTTCAGTGGTTGGATTTTTAGATGAGGGATTATTGG | TTCAAGAATGCGGGCAATGTGTCAATTGCATTAC | 201346 |
| 201347 | GTTTCAGTGGTTGGATTTTTAGATGAGGGATTATTGG | CGGCTGGTATTACAAGGATGCAAGACACATGGAT | 201417 |
| 201418 | GTTTCAGTGGTTGGATTTTTAGATGAGGGATTATTGG | TGTAGATGATGGTTTTCAAGTGGCAATTGTTCAAGA | 201490 |
| 201491 | GTTTCAGTGGTTGGATTTTTAGATGAGGGATTATTGG |  | 201527 |
| Legionella pneumophila subsp. pneumophila GCF_001592705 | | |  |
| RefSeq :NZ_CP012019 (chromosome circular) | | Number of repetitions :44 |  |
| Position | Repeat | Spacer | Position |
| 2540371 | GATTCAATCCGCACGCCCGCACGGGGCGCGAC | CGTCTACTTTTCTATTTGACACAATTGCTATTCCT | 2540437 |
| 2540438 | GTTTCAATCCACGCGCCCGCACGGGGCGCGAC | CCTATTGGCATTGACGACCTAATCACCAAACTCAAA | 2540505 |
| 2540506 | GTTTCAATCCACGCGCCCGCACGGGGCGCGAC | GAATGATGATGTGCAAGCTATTTTACCTTGGGAT | 2540571 |
| 2540572 | GTTTCAATCCACGCGCCCGCACGGGGCGCGAC | CTATATTGAAATATTTTTCTTCTTTTTCTATACCTAT | 2540640 |
| 2540641 | GTTTCAATCCACGCGCCCGCACGGGGCGCGAC | AGTTAAATACAAACGCGTAAAATCTAAGTTATCT | 2540706 |
| 2540707 | GTTTCAATCCACGCGCCCGCACGGGGCGCGAC | ATGAAAACTCTAGTTTTTAATGTCTTAGACGACG | 2540772 |
| 2540773 | GTTTCAATCCACGCGCCCGCACGGGGCGCGAC | ATTTTTACATTCATTCTATAAGTTTGCGAAATAT | 2540838 |
| 2540839 | GTTTCAATCCACGCGCCCGCACGGGGCGCGAC | GACAGCATTGCGTCAAGAGTTTATTGCTTTAGATAA | 2540906 |
| 2540907 | GTTTCAATCCACGCGCCCGCACGGGGCGCGAC | TTCTTCAATAGTCATTACTTTACCTCTCTTTAGAT | 2540973 |
| 2540974 | GTTTCAATCCACGCGCCCGCACGGGGCGCGAC | TCCAAGGCTTCTTTTTGTAACCCTTGTTTTTGCTT | 2541040 |
| 2541041 | GTTTCAATCCACGCGCCCGCACGGGGCGCGAC | AACAACCCAATCATTATTTTCTGAGCCTATAAAAT | 2541107 |
| 2541108 | GTTTCAATCCACGCGCCCGCACGGGGCGCGAC | ATGCCCTATCACAAGAGCTATTTCATCATCATTTTCC | 2541176 |
| 2541177 | GTTTCAATCCACGCGCCCGCACGGGGCGCGAC | CTAAGACAACTATTCTTAATTCACATAGAGACACAA | 2541244 |
| 2541245 | GTTTCAATCCACGCGCCCGCACGGGGCGCGAC | AAATTATTAGCAATTCCCTGATCCAACTAGGCCAT | 2541311 |
| 2541312 | GTTTCAATCCACGCGCCCGCACGGGGCGCGAC | GTAAATCATATATAAATAGTGGTGAGTGGTGTGA | 2541377 |
| 2541378 | GTTTCAATCCACGCGCCCGCACGGGGCGCGAC | ACCATACACTAAGAATAGACGTTACTGATAAACTA | 2541444 |
| 2541445 | GTTTCAATCCACGCGCCCGCACGGGGCGCGAC | AGGACCTATAGCCACACCTTGGGCGGGCCATAAA | 2541510 |
| 2541511 | GTTTCAATCCACGCGCCCGCACGGGGCGCGAC | CAATAAATCTAGTCCTATCCCTAACTAATTGTAATT | 2541578 |
| 2541579 | GTTTCAATCCACGCGCCCGCACGGGGCGCGAC | CCCCTATGACATAAATGTACCTGAGATTTACGAA | 2541644 |
| 2541645 | GTTTCAATCCACGCGCCCGCACGGGGCGCGAC | AACGACAGCGAAACATGTTGATCAGCCATACAAAG | 2541711 |
| 2541712 | GTTTCAATCCACGCGCCCGCACGGGGCGCGAC | GCATCAGGACGGGCTTTAAATAACAATCCAGTTTC | 2541778 |
| 2541779 | GTTTCAATCCACGCGCCCGCACGGGGCGCGAC | CACCTGTAAACAATCCTTTCAAACTTCCTAAAACAA | 2541846 |
| 2541847 | GTTTCAATCCACGCGCCCGCACGGGGCGCGAC | ACTACAATATAAATCACATTTACTATTTTCTTGA | 2541912 |
| 2541913 | GTTTCAATCCACGCGCCCGCACGGGGCGCGAC | ACCATGAAAGCATTGAAGTAACCGCTTGCTGCAATCG | 2541981 |
| 2541982 | GTTTCAATCCACGCGCCCGCACGGGGCGCGAC | CTGTCCCTTTAACTGCTGCCAACATCATTGCCAT | 2542047 |
| 2542048 | GTTTCAATCCACGCGCCCGCACGGGGCGCGAC | TCTATATGCGTACATGTTGCCCTTACTGTGGCATT | 2542114 |
| 2542115 | GTTTCAATCCACGCGCCCGCACGGGGCGCGAC | TCGACTTGGCCTTATCCAAAGCTAGATTTATAATT | 2542181 |
| 2542182 | GTTTCAATCCACGCGCCCGCACGGGGCGCGAC | ACTAAACACGAAAAATGGGCAATCGAATTAAGAA | 2542247 |
| 2542248 | GTTTCAATCCACGCGCCCGCACGGGGCGCGAC | TCGCTAAAAGCCCCTGCCAAGATTATTAATGTATTA | 2542315 |
| 2542316 | GTTTCAATCCACGCGCCCGCACGGGGCGCGAC | CCATTTTATAGCAGTAAGTGATACCAAGTATCCG | 2542381 |
| 2542382 | GTTTCAATCCACGCGCCCGCACGGGGCGCGAC | GTTGGTTTCCCTCACAGACAAATATCATTATACAT | 2542448 |
| 2542449 | GTTTCAATCCACGCGCCCGCACGGGGCGCGAC | ACCATACACTAAGAATAGACGTTACTGATAAACTA | 2542515 |
| 2542516 | GTTTCAATCCACGCGCCCGCACGGGGCGCGAC | AGTAAAGTTTGGTTTACGTTGTGAGATTATTAT | 2542580 |
| 2542581 | GTTTCAATCCACGCGCCCGCACGGGGCGCGAC | ATGCATTGGTTCTATACGAATTTCATGGAGTACGCT | 2542648 |
| 2542649 | GTTTCAATCCACGCGCCCGCACGGGGCGCGAC | GTAAGTGCTATAGCAATGTGGTTTGACAAGAACAT | 2542715 |
| 2542716 | GTTTCAATCCACGCGCCCGCACGGGGCGCGAC | CTGTCCCTTTAACTGCTGCCAACATCATTGCGAT | 2542781 |
| 2542782 | GTTTCAATCCACGCGCCCGCACGGGGCGCGAC | AGCATCGTTGATTACATTAATAGCAATGGCACATGG | 2542849 |
| 2542850 | GTTTCAATCCACGCGCCCGCACGGGGCGCGAC | ACCTTTGCCAAGAGCAACGTTCACCATGAAAGCATT | 2542917 |
| 2542918 | GTTTCAATCCACGCGCCCGCACGGGGCGCGAC | CCTGTGATCCCGGTTACGGCTGTGCCGTTAAAAGT | 2542984 |
| 2542985 | GTTTCAATCCACGCGCCCGCACGGGGCGCGAC | CTGTTGTTTTTTGACATAGAAACCAGTTTAATT | 2543049 |
| 2543050 | GTTTCAATCCACGCGCCCGCACGGGGCGCGAC | GAAACAGATGATCAGGTGATTAAAAACGATTTAAA | 2543116 |
| 2543117 | GTTTCAATCCACGCGCCCGCACGGGGCGCGAC | ACCCGGACTGGGTTAAAGCCTATCACCGCGAGATG | 2543183 |
| 2543184 | GTTTCAATCCACGCGCCCGCACGGGGCGCGAC | CGGAAAGATGAAACCTAGCCAACCTATAGACTTAA | 2543250 |
| 2543251 | GTTTCAATCCACGCGCCCGCACGGGGCGCGAC |  | 2543282 |
