# Supplementary material for: A new method for quantitative detection of Lactobacillus casei based on casx gene and its application
Source: BMC Biotechnol. 2019 Dec 10;19:87. doi: 10.1186/s12896-019-0587-6 (PMC6902566; doi:10.1186/s12896-019-0587-6)
Supplement: Supplementary file 2 — Additional file 2: Table S2. Flanking sequence of CRISPR region. [file 12896_2019_587_MOESM2_ESM.docx]

Table S2 Flanking sequence of CRISPR region

| Legionella pneumophila subsp. fraseri GCF_001886795 | |
| --- | --- |
| Left flanking sequence : Till position 200766 (length 3000 bp) | Right flanking sequence : From position 201527 (length 3000 bp) |
| ACACACACATTGAGACAATCAGGTTTAACATATGGTATTCAAAAGTAACGCCCTGCAATCATTGCCGGACGGGTCAGCGCATTGAATATGGGGCTTATTGCCAAATCGCTCACAAGAATCTCCTATCCAATGAGGGTTGAGTACAGGGAAGATCCACCAAAGATGATCCCCATTCCTACAAGAATCATCACAAAATATTCCTTGGGAAATTTTTGACGTGCCAGACAGAGGTAGCCTGCAATGATGATGCAAAACACCCCTACGGTTCTTGCAAGCCCACCCTGAAGGTAATTAATGGTTCTGTTGATGATGCTTTCTATCGATTGGGCATGGGATAGTGATGGCAGTAGAGTACAAACAGCCACTAGATAGAGATCGCTAATCGATAGACTTAAACGGTTAAATTTGCTCATAGTGCCACCTTCTGCATGGAAGATTCAGCCTGCTCCTTCTGGAGTTTTTTCAGATAAACTTCTTCGCGGTCAATATCGATATGCTGAGGGGCTTTAATGCCAATGCTGATGATGTCGTCATCGACCTTGAGCACCTTCATTTGAATCAAGCCCTTGCCTATTAAAATCTGTTGCCCTGCTTTTCGTGTTAAAACCAACATGATGTTCTCCGGTTATTTTGCTAAAAGTTCCTTGCACTTCTGCAAACAGGAACTCACAACAGCTTTGTTTGCAGGTTGATACACATCCTTAACCACATAAAACGACTCTTGCTTCTTGATTTGCCTGCAGCAATACAGCATTTGCGGCAGCGTAGGCGGTAACTCGTAAAACTCACGGCAATTCAAAGTCGCCTTGGTCAGTACCGCATCTGTAAAATCAGCTAATGCCTCTTGCCATTCTTTCTTTGCAAACTTCAAAAACACTTCATCCTTAAACTGGCTTCTCCAAACATGGCCATAAAAGGCCGCAAATTTAGAGAACAGCAAATCAATACGTCTCTCAGCGTTGAAGGTATCTTTGGAGCTGGATGACATTGTTATTCCCTCGCTCTTCGACATCACAGGGTGGGCAGAACAAGTCTTCTGTGGTGTGACGTTTACGAGTATTTTTTGTATGCTCTGCATGGTGCTTCTCCTGTAATGCATCCGTAGATAATTCATCCTCCCAGCAACGTTGGGCAAGCCAATTTGCCGGGTATTTCCAAGGCGGCACCCATGTGCCTTGTAGTTTCATGGTGTCACGATGATTAATTTGGGCATTCAATGCGTTGATGATGGTGTGAAATAGCTCAGCGTTAGGTTTTAGCTCTTCAAAAACGGCCTGTGCGTTGTTCTTTGATTTTTTCTCAGGATAAAGCGACCAGAATTTTTCGAATTGCGATAATAAATAAATATAATAATTATCTTTATGAGGTATGGCGGCTTTTGAGGGTTCCACTGTGTCGGCTTTTGGTGCCTCAACCTCGGATAATCCAGTATTTTCAAGGGATTCCCCACGCGGCTTTATGGTGGCTTTCTGTGGTGGGTTTATGGCGGCTTTATTTTGGACAGAATAATGCTGTGAAGCTAAAAGACATTTTAAAATCAAATGCATCCCTTCGGATTGCACTTCGATTAAGCCCGCACGAACCAATCCTGAAACTGCACGCCGAATCTGATCACGCGAAAAGCTCTGGCTTTTTATGCCTTGATGCGGCTCAACATAAAGCTGCTCAGAAATCGACTGATGACTAATGCGCCTTTTGATTCCGACTATTCGGGTTTTAACGTCCATATAAGGTCGTATCCCTCTTAAATAAGTCAGTTGCTGAATATGGGGTAAACCACACATTGCCTCTAACTCATCACCATTAATCACAAAATCCATCATGGGTAATATCCCTACTCATCCTCGTTTTTATTTCCATAATGTGATATCTTTATAAAAATAAAAGATTATTATTCACGATATGTGAATTTATAAACTAATTTATATCACGAAAAGTGATATTGCAATAGTTAACTCTCATTTTTTAGGGGTAAATTTGACTGAGCTGAATATCAAAAAGGAAATTGGCAAACGTATTCTTGAAGCACGGAAGGCTAAAGGCTTGACGTTAAAGGCTCTTGGCGAGCTTGCTGGGGGATTGAAACAAACGCGCCTGACGAACTGGGAACAGGGTGTGCGTACACCCGGGCCGGAAGAGATTAAGTCGCTTGCGCAGGCATTGGATGTTTCGCCTGCTTACCTGATGTGCTTGTCAGATGAGAAGCGGTTTAAGGAAGTACAAAGCCCGAGCCAGTTAGTTCCATTATTAACTCACTATCAAGCTTGTGAGGCATTATTGCATATCCAAACAATCAGAGAGCAAGAAAACCCGAATAATATAACTCTTATTTCTGTGAGCATGGCATTGTTACCTGTTTTAAGCGCAGACGCGTTTGCATTGAAAATGCTGGATGACAGCATGATTCCAGAGATAAGAATTAATGATATACAGGTGATTGACCCAAATGCTCAACCCAATCCGGGAGATTATGTTGCTGTTAAAGTCAGAGGTAAAGAAGAGGCAATTATCTGTGTATATAAAAAATTGTCCTATACATCCTCACAATTCGAGCTTTTAACGCTTAATGATAATTGGCCAGATATTAAAGTGAGTGATATTGCTGAGGTTAATATTCTTGGGAAATTAGTCCTGAATATTAGAACTTACTGAAATAATTATCTAATAAAATAAATTCACTAGTATTATATTCAGTTGAGATGTACAAAACAAAAGACAATCCAGTGTTGGGCATGGTTTTGAAAATTAAATTAAGAAAATTTGTTACAAAGATAATCTGTAAAGACAGGTATTGTCAGAAAAAGCAACAGAGTAAAATAAAAGCACTTAATAATTATCAAGTTAATTATTAAGCAAATAAGATGAAAAACAAGCAAACATAACGGCTGTCGTAACTCCATGAAAGTGTCACATTCATGGAGTTACGACATGTTTAATTATATAAAATAATAACTCCATGGAGTTTTACTATAATAGTATACAGAGAATAAAGTATGGAGT | AGCAATTTTAACTCTTTGTTTATTTTAAAGAAACACGAGGAATTAAAAGTGGTTTTCTTCGAAAAATAGCAAATTGCATTATTTAGACTTGATTTTTCGAAAAAAAAGGCAATACTTGAATTAGAAATCATCTAAATTTCGATACCCTGAAATCAACAAAATTAAAGATTGAATCGTTTTTCTATGCTCGTATTAATAGCGAGCATATAACGATTTTAAAATCTATCGTTACAGTGATTTAGAAACTCACACTGAATGCATTGAAGTCCATTAGCAGTGCTGTATGGTAATGTTTCTGTGTCAAAAATCTTCTTTATTTTAGCATAAATTTCAAGCACTTTGAACTTTGATTTTTCGTCAATTTTTATTAATTTGGCTTTGGCTTTTTCACCAGTAATAATATAGCCTCGGGTACATGATTTTTCAAAAATCAATTCAGAAAGCATTCCATATGCGGTCAGTTGCATCTTTTGACCTAAACTAATTTTTGATGCAAAAGATTTTAATTCAATAGGGATAATTTCTAGTTCATCTTCAATGATTCCATCACAAATCCCATAAAATGGGTAAGATGGATGCTCAAGCATAGTGTTTTGTCTGAGTATAGCCTGTTCTGAAATTGGCTTGAATTTTGAGAGAGTTCTTCTTTTATCTAATATCTGTTGCTTATGTTGATATTCCTCACCGTATTGCACCCAGCCAGGGTAGGCTACTTTCAGGCCAATTACCATATGGTAGTAAACAATGCGGGGACAAAAGCAGTACTGCCTTATCATATTTACAGGCAGTTTATACGATGATGTGTCCATCCGGTTTCCTAAGTTCATCCAGTTCAAGATTAATGAGGTTTACTTCATATTCTTGAGTCAGCTTACAAGGTGTAATAAAAGCAGTATCTTGTTTGGCAAGTTCTTTTTTTAGAACATTCGTTAAATATCTTTTTTCAGGTTCGTTTAAATATCCCCAAAATACTGATTTTTGAACGGGGGTTAAGCCAATGTCTTTAAGCCTTTCGTAAATGGTACGCCGGATGCTGTTGTCCCTGATGTCGTAGGAAACTATGTATTCCTTGTATAATCTTGTTTGCATTTTTCATTACCATTTAAATAAGTAGCCTTTATACTTTCCTTCACCGGAGAATACAGCTGACAGGTGATAGACTTGTCTTTGAATGATGCTTTCAAGGGATATTCTTCGTTTGTGGTATGGCAGTGGCTTTGCGTATAAATTTGCCATCTCATCAATTAGCGCTTTTTTCAAAGCGGGGGTTAGTTCATTCTGTTTTTGGGCGCGGGCGCGGAGCTTAAAAATAATTCTATCGGCACACCAGGGTCGAAATTCTTCCATGAGATCGAGTACTAACGATGGTTTCCCTGGTTTGGATGTATGTAAAATTCCAGCATATAGCTCTAGTCCAGCATTAGTTATGGCCTGCCATACCCAACTGGACAAAATCGAGTAGGCATAATTTAACATAGAATTTGTAATGTCTTTGTTTACCCTTGGGCGTCTCCCTTCGAAATCACTGCATACCAAATTGACCTCTTTAACTGTTTCCCAATATATTTTGGCAGCACTTCCTTCAAATCCGAGTAGCGTAAACCGCCAATCCTGAAGTTTTATCCAATTTGTTTCTGTTATGTTGGAAACAATGCCGGCTATTTTCTGGGATGCTGTGTGAAGAATTATTTTCTCATTTTCAGGCCGGTTTTTAGCTTTTGCAAAGTATTTCAGAACGGCGGACTGGTTTTTAATTTTCGCTAAAATGAATCGAGCAGCCATGTTGGATGCTTCTTTGGTGTATATGTATTTGAACTGGTTTTCCCGTACTTTGGCTGTGCGATGGAGTCCACCCTGAAGGCTACAAACTGCTTTACCCGAATAATCCAGAATGAAAATTTGTATTCCTCTCATTGCGCAGGCAACAAGAAGATTTGAGGTCAAAGTGACGCCTTGTTTGGCGATGCTTATGGTTTTTAAACGGTTGAGCGTTCCTTCAAAAACCATGTGATCTTTATTAAAGACAAGGATTCTTTCGCCTTGCAACTTACAAGAGCTGCCTTGTTCAGAGAGCGTGAGGTGTTTCATGGATGTTTTTTCAGTTGAGTGACATAGAGTTTCTGTATCCATTGTGGTGTGGAATCGGATTCAAACTCATAAGTCACAGTTTTTCCAGTTGATACAATTTTCATTTTCCCATCACGTGGTTTTAATTCATTACCGAATAATTTGTTTTTACAAACAATTTCATTGGGCATGTTTAAGGGATCATCAATTGAATCGGAGGGTTTAATCATTAAAGCCTCGTCAATGGTTTTAATAAAATCAGCTAAAGATTGGGTAATTCGTATATAGCGCCTGGTTTTGGAGTGGGGCTTCATTTCGAGTTTTATAATTTCTGGTTTGAAGGTTGAAACAGGCAGAGTTAGCCAATTATCGAAAGCAGCATACGCCTGTCTCTCGGAATTATTAATACCGTCAATTGTTGATAAATTTCTGGTTTTATAAGCATCCATAAGTACTTCTTGTTTGACCAGTTTACCTTCATTAATTTGAATTCCCATGCTAGGTGTGTCATCAATGGTCTGTAGTTGGTATAAGGGTTGTCCTTTATTATCTTTGCGCCTGATACGCATCATGGTGCCTGCATTACCAGGTATTACTGGCAGTGAGAATACTTTTCGAACGGTTTTGTGCTTTTGTGGCTTAATGGTGTGGTCATTGTTTGGAATGTCCGAATTTGGGTTGTTTTCAGAAAGAAAATACTTCTTGATAAATTCGTTAAACTCTTTTGGGTTGGGGTTAGGGTTTGCTTTTAGTGCAAGAAAATTTGGATGGTTAAAGAGCCTCTCCCAATCTTTGGTAGCTGGCAAAGCAATTGTATGTTTAAAAGTGCCTAAAACATTTTTCTTGGAAGATTCGAATCTTACGCTTAGAACAGGCATAGACTCTTTTAATATCTTCACAGTAATGGATTCCTTTTTAGTGTAGTAACGTAGG |
| Legionella pneumophila subsp. pneumophila GCF_001592705 | |
| Left flanking sequence : Till position 2540371 (length 3000 bp) | Right flanking sequence : From position 2543282 (length 3000 bp) |
| GTTTCCAGAAAAGCAGCAGAGAGAACGGTATATTTTGAATCCATTTTAATTTTCTTGGGAGGAGTAATTGGTACTGGTCATCATTGGTACTGGACTGGGACTCCTGAAATGTGGATTCCGCTAGGCTCCATGTTTTCCTTTATCGAAGTCTTGCCTCTGGTTCTTCTTATTATTGATGCGATTGAGCATCGACAATTGATTAAAAAGCAGGGAACGTTTACCTACAATCTCGCTTATCTCTACATATTGGGAGCCGCGTTCTGGAATTTTGTTGGTGCCGGTGTATTCGGAGGAGGTACACTGAATGCGCCACTGATTAATTACTATGAGCATGGAACATTCTTGACTTTAAATCATGCCCATACTGCCTTATTTGGCGCATTTGGGCTTCTTGCTCTCGGGTTAATCTATTTTTGCCTGAGATATGCTGCTGGTGAAGGCAAGACATGGAGTGATCGATTGGGTGTTTATGCCTTTTGGTTATATAATGCAGGGCTCTTTTTATGGATTGTCCTGAATTTTTTCCCAATTGGCTGGTCGCAGTTAATGGCAGTATATGAACATGGACTATCCTATGCCCGAAGCCTTGAATTCTATAACACCACCTTATTATGGCAATGGCTTCGTTTTCCAGGGGATGTCGTATTTGCCTTGGGGGCTCTTCTTATGGCTTATGATTTTATTGTTAAGATTGGACCTTTTTTCCCGAAATTTGCCCGTAATCGTCGCTTTATAGCTGGCCCTCCAAAAGCAACAGACCCAAGCTTATGACCAATGATAGCAAAGACAAAGCCAGTATATTTTTTTGAAAAATTATTTTGAGAAACCGAGTTTAGAAGTGAATATCGACACCCAAATGGTGGGTGTCGGCTTGCAATCAAATTTAAGGTAAGAGATCAGCCTCTACCTGGAACGAGCGTATTTGGTTCATCTTGAACGACAATTGCTTTTTTTGGTTAAATTACACTTGCGCCAAATAAGGCCGGTCAAATTCTTTAGCTAGCTCAGCCTCATCTTCTTGTTCCACAGGCAAGAAGCCTGAGGTTTGCGCATCCCACATGGATTTATATAATCCATTGTTTTCTATCAACTCTTGATGTGTGCCATCCTCTACTATTGTTCCTTTATCAAAAACCAGGATTCTATCCATGTTAAGAAGGGTTGAAAGTCGATGCGCTACAACGATAGTTGTTTTTCCCTCGCATAGTGGTTGCAAGGATTCTTGAATGCATTCTTCAGTTAAAGAATCCAGTTGTGAAGTGGCTTCATCTAAAATCAATATAGGAGTATGATGCAAAAACGCTCTTGCTATAGCGATTCTTTGTCTTTGCCCCCCTGACAACTTAGTACCCCTTTCCCCTACTATAGTATTAAAACCCTGAGGCAAGTTCTGAATAAAGTCCATGCAATTTGCTAAAGAAGCTGCTGTTACTACTTCATCCCTGTCAGCATCCTCTTTACCATATTGAATGTTTTCTAATAAGGTACGATGGAATAGATTGGTATCCTGAGGAATCATACTGATTGATTTTCTTAAAGAATATTGCGTGATCTCGCTAATGTCTTGATTATCAATCAATATCCTTCCGCTATCTACATCAAAATAACGGAGAATTAGATTGATAAATGTTGTTTTGCCTCCCCCGGAATAGCCTACAAGCCCAACTTTTTGTTTAGCCGGAATAACCAACGATTTATTGGTAAATAATTCCTGACCATCTCTATGGCGAAAGGTCACATTTTCAAACCTGATTTCACCATGAGTGACTTTCAACTCTTTTGCACTGGCTATATCTTGAACTTCTATAGGTTGAGATAATATGGCAAGCGCCTGCTTGACTATCCCTATTTCGACAAACAAATCACAAAGCGAATTACTTAGACTCCACATTTGATTCATAATGGCAAATGATGTTGCGAATATAAAGACCACGTCACCTGTGCTGATTAAGTTTCTCTGCCAGAAACTGATGACTGAATAAATCATGGCAATTAACATAATACTGACAGGAACATCGACTAAAAGGCGAAAAAAATTGATGAAAAGAACCAGACGCTTATTACTTTGAACCTCTTTATCTTGTGTTTTCCAAACGTATTTTTTTTCATACTCATTTTGGACAAATAATTTCACGGTATTGATATTATTTAAGGAATCAACGATCTTTCCCTTTAAAATACTTTTATCTTCCGCATTCACCCTGGCAAGCTTATTGATCTCTTTAGCTGAATAAAAGCTGATAAATAATTGGATTATGATCCAGCTTAACAAAATGAATGCAAACCAGGGATTAATGGTTAACATTAAAACGATTGAAACTACAATTGCCGCAAAAGTAGTGTTTACATTACAAGATACGATTTTACGAATGGCTTCCAATGATCGAGGTATATCACTTATCTTATTAGCCAGGTTTCCCGCTAATTGATTAGAAAAAAAATGATGGGAATGCTGGATTAAATAACCGAATAGGGTCATGCGGATATTGGCCTGAAATTGAGGAACAAGGTAGGCTTGCCACCAATTATGCAAGCGCAACACCAGAATCACACCAAACCAGGCAACTCCACCCAAATACAAAGCCGGTGCGATGTCTTGAAATATGCTTTCTTTTCCCCCTTTATGGTTTGTTATCCCATCAATAATCATTTTTAAAGCATAGGGAATCACATTCGTTTCAAGCACCATCACCACTGGTGCTATGAAAAATAAAGCATAAAGCCAGGGACTTTTTTTTATAAAATGCCAGGAAAATCTCAGGATAGTCTTAGGCAATTGTTGTAAGTCAGACATATTCACATCCTTGTAAAAAATCTGGAACTTCTTCTTTAAGATATCAAAGTCAAAGATAAAGACAAAGAGCTTCTGGCTAAATATACCTAAAATCGATATGAAATGTTTGTAACAATTCACATCAAGTTCATTTTAATTAAAATTTATTCATACTATCTTTGCTAAACGCGACGCTTTTGTTCACTTAAAAAACTCATGCTACCCGT | AGTATGATTATAGCATATTGAAATATATCGAAAAAAATGCTAAATATCGCTAATAATAATAAAAAATTATATTTATTGATTAGCTTGATAAGCACACATAAAAAAAAATATAAATATCAAGTGGTTATAATCCGATAATTGACCGGTAAAAATATGAGAACTTCAAGTTAGCGAATTACAGTATCAGTGGATCTCTCAAGGGGTCGATGACCTTATTAGCTCCAAAATGTTCGATTTTATCCTTCCCTTTTTTGCCTAAAAAATAAAATCGTAAGCTATCAGCATCAGGATCAAATAACTCTAAAAGTGTATTTTTTAACGCAACGAACCGATCAGGTTCAACTTCGCATTCAAATACAGAGTTTTGCACTCTAAAACCATAATCAAGACATGTTTTTGCAATATTCCGTAAACGTTTCTGCCCACCAGAAGTTGTTACATTAACATCATAAGTTATTACTACAAACATAATTACTTCACTAAATAAGGAGTATAAAATTCTGTATCGCCCCGCAAATGACGAGCTAATAATAATGCCTGACAGTGTGGTAATAATCCTACAGTAACCTGTTCCTTTAAATAAGGATGTATCACCTCCATTTGCTTGCGCTCTTGATAAGATACTAACAATGATTTACGAGCGTTCTCAGTTAAACGTACTGCTCCACTTGCCTCTGTCACAAAATCGCTTAATTGTATTTGCCGACGATTGATTAAAGTCAATATAAAACGATCGGCCAAAGGAGCACGAAACTCCTCCAGTAAATCTAAAGCCATACTTGCCCTACCTGGCCTATCTCTATGTAAAAATCCGACGTAGGGATCAAGGCCAACCCCTAATAATGCAGAAATACATTCCTGAGTTAATAAAGAATAAATAAATGATAGCAACGCATTGACAGGATCAGTAGGAGGTCGACGATTACGGGATGAAAATGTGAACTTTTCTCCATGTATTAATTCACTAAAAACACCAAAATAAACCGCAGCTGAATCGCCTTCTATACCCATCAACTCTTGAATATTTAATGCACTTCGAGATCGATGTAAATTTCTTAACAACTTTTCAGCCGAATTTTCAAGCAATGCATTTTCACCATGATTACGTATTTCTCGCATCAAGACTGAACGGCAATTAGCAATTTTTGCCCCTACCATAATGCGTGCAATAGACAATGATTTATCCTGATGATCAGCCCATCGATACTGGGTTCGCCTTAAGAGAACATTGCCAGTCTGCTTACCTAAAACGCGCGCAAAAAACTTGCCATATTCAGAATAGAAAGATAAACCAATGCCTCGTTCCCCACAAAATCCCATTAATTGAGGTGAAACTGAAACTTGTCCAAAACATAGAATATTCCCTATGGTCAAAGCAGGAAACTGCCCTAGTTTTTCTTTCCCTAACATGATCACAATAGTTTCACGATCCTTATGTAAATAGGTTTCCTGGCGTGTTACGTAGAGTGTATTGAGTAATTTTTTCATAATAAATACATTGAGTTGACATAGATTTTTGAATGATCATGCTGTAGACATTGAGGCTCACATAAATCGATGAGTGAACAAGCATTGCAACGTTTGCTAAATAGCGCACGCGGGGTTATGCCTGACTCTATCATAGCATGAGCAGCAGCAATGATTTCCTCTGTTTTTTCACGTAATTGTTTATCAATTGGTACAATCTCACGATGGCGTATTTGCCAATACCACAACGCAGCCTCTTTAATTTGAACATTACGCATTTCTTCCAAACACAAAACTTGTGCACATAATTGCGCTTTATCCCATATTTCTAGTTTGGGTTTACCGCGTTTATATTCCACGGGGAAATAACTGACAGGAGACTTACCCTGTACCTCCAACAAATCTAATTTCCCCCTTATACACAAACGATGCGAAATAACCATCACACCTCTCTCTGAACGTAGATTACCACGTTGTTCACTGCCTCCAGCATCTACACGAGCATGTAACGCTCTACCTTCAGCTGTCCAAAAATTTTCTGACCATACCTGTTCTATATGAATCAAAGCGCATTGCCTGGGACAGTAGGCATAGTGCTGAATGGCAGAAACTAATAGATAGTCATCTTCAGTCATATCAGCCAATTATCCGTTCTAACATAACGCCTTGAGGAAAATTGCTTGTATCAACTTCTACCTTATAATCATCAAAAGAGCGAGCAGGGCCTTCCGTTTGGCGTGATATTTTAATCCGCTCAAATAAAGTATGTGCTTGAGCATTACCTAATTTGGATTCATGTTTAAAGACATATAGCCCTCGAGCCGTCATTTCTCCTCTTGCGGCCGATCGGTCATGTTCAAACATGTTTTTGAATGATTCAAAAAGCAGCTCCAAATCATCTTCATTGAAATTGGTCTGCTCAGCAAGGGGTGCAGAAATAAAACCGTGCATTCTATATAACCCATAAGGAATAGTAAATTTACGCCCCATTGTTCGATTGTCGCCCTCCTGATTTTCGGCTTCATCTTCTGTAGCCACTGCCATACGTGTAATAGTATGCTCGGAGGTTACAATAGGTTCAACAGAGCGTGCGAAAGTCAACTGCACAGGTCCTCTGACTTGTCCGGCATTTATTCCTGTACTCATGACGGCACCAAAAGTACGCACATCATAAAAGTTCAAACACATCCACTGACGCGCTTCTTCAACTTTCCCTCCCCCTTTGCGCTTTTTATTATCTACCTTGAGTAGTTCTTCTTCTCCTATCCCAATGTATGCCCGTTCATTTTGTTTATTAAGAATAGCTTTTTCCCGAACATAGATCTCATAACGCATCTGATTTTTATCAAGCGGTTGGGTCAATTCCATTGGATTTTTGACTAAAGAAACATAATTCCTTATCTTTCTTTTCAAACAAACATCAGTGACCAATCCGCACCCAGTCTCAACATCAATGCGTGGTAAATTTCCTGCATCCGGATCTCCATTAGGATTCCCATCTTTAACATCAAAAATGAATACTAAATCGTAGCG |
