# Supplementary material for: A new method for quantitative detection of Lactobacillus casei based on casx gene and its application
Source: BMC Biotechnol. 2019 Dec 10;19:87. doi: 10.1186/s12896-019-0587-6 (PMC6902566; doi:10.1186/s12896-019-0587-6)
Supplement: Supplementary file 3 — Additional file 3: Table S3. Specific sequence selected from sequence alignment results. [file 12896_2019_587_MOESM3_ESM.docx]

Table S3. Specific sequence selected from sequence alignment results

| Position | Sequence(256bp) |
| --- | --- |
| 2540931-2541187 | ACATGGACTATCCTATGCCCGAAGCCTTGAATTCTATAACACCACCTTATTATGGCAATGGCTTCGTTTTCCAGGGGATGTCGTATTTGCCTTGGGGGCTCTTCTTATGGCTTATGATTTTATTGTTAAGATTGGACCTTTTTTCCCGAAATTTGCCCGTAATCGTCGCTTTATAGCTGGCCCTCCAAAAGCAACAGACCCAAGCTTATGACCAATGATAGCAAAGACAAAGCCAGTATATTTTTTTGAAAAATTA |
