# Supplementary figures and images for: A new method for quantitative detection of Lactobacillus casei based on casx gene and its application
Source: BMC Biotechnol. 2019 Dec 10;19:87. doi: 10.1186/s12896-019-0587-6 (PMC6902566; doi:10.1186/s12896-019-0587-6)

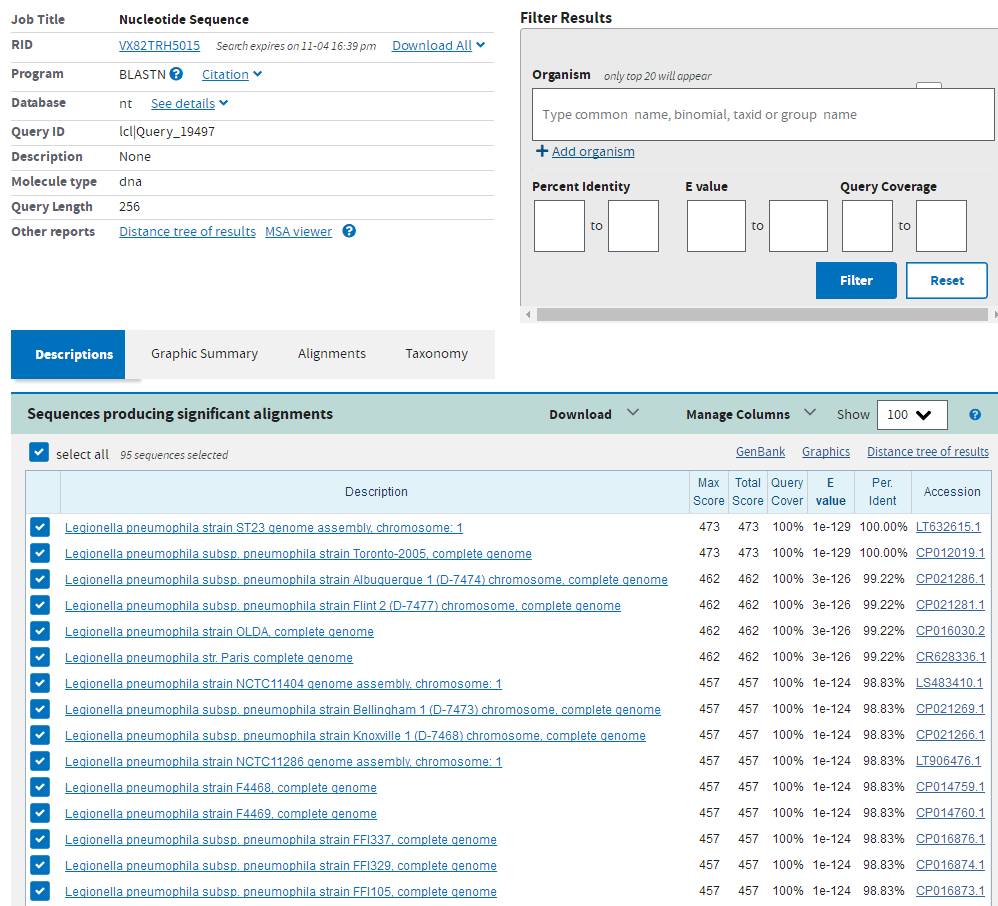


Figure S1 The result of sequence BLAST

Supplement: Supplementary file 4 — Additional file 4: Figure S1. The result of sequence BLAST. [file 12896_2019_587_MOESM4_ESM.docx]
